# Supplementary material for: Exploring the relation between Interleukin-6 and high-sensitive cardiac troponin T in asymptomatic hemodialysis patient: A cross-sectional study
Source: PLoS One. 2024 Jan 25;19(1):e0296965. doi: 10.1371/journal.pone.0296965 (PMC10810457; doi:10.1371/journal.pone.0296965)
Supplement: S1 File — (PDF) [file pone.0296965.s001.pdf]

-Najah National University
culty of Medicine & Health Sciences
nstitutional Review Board

جامعة النجاح الوطنية
كلية الطب وعلوم الصحة
لجنة اخلاقيات البحث العلمي

Ref: Med. June. 2022/8

IRB Approval Letter

Title of Research:

Interleukin-6 in hemodialysis patients and correlation with cardiac troponin: A cross sectional

Submitted by:
Leen Ibrahim, Katreen Yaseen, Leen Abbas

Supervosor:
ZaherNazzal, Zakaria Hamdan, Yahya Ismail

Approved:
13th June 2022.

Your Study Title **"Interleukin-6 in hemodialysis patients and correlation with cardiac troponin: A cross sectional ."**reviewed by An-Najah National University IRB committee and was approved on 13th June 2022.

Hasan Fitian, MD

IRB Committee Chairman

IRB
